# Mild hypoxic-ischemic encephalopathy (HIE): timing and pattern of MRI brain injury

**DOI:** 10.1038/s41390-022-02026-7

**Published:** 2022-03-30

**Authors:** Yi Li, Jessica L. Wisnowski, Lina Chalak, Amit M. Mathur, Robert C. McKinstry, Genesis Licona, Dennis E. Mayock, Taeun Chang, Krisa P. Van Meurs, Tai-Wei Wu, Kaashif A. Ahmad, Marie-Coralie Cornet, Rakesh Rao, Aaron Scheffler, Yvonne W. Wu

**Affiliations:** 1 Department of Radiology and Biomedical Imaging, University of California San Francisco, San Francisco, CA USA; 2 Department of Radiology and Pediatrics, Children’s Hospital Los Angeles, University of Southern California, Los Angeles, CA USA; 3 Department of Pediatrics, University of Texas Southwestern Medical Center, Dallas, TX USA; 4 Division of Neonatal Perinatal Medicine, Department of Pediatrics, Saint Louis University School of Medicine, St. Louis, MO USA; 5 Mallinckrodt Institute of Radiology, Washington University School of Medicine, St. Louis, MO USA; 6 Division of Neonatology, Department of Pediatrics, Vanderbilt University School of Medicine, Nashville, TN USA; 7 Division of Neonatology, Department of Pediatrics, University of Washington School of Medicine, Seattle, WA USA; 8 Department of Neurology, Children’s National Hospital, George Washington School of Medicine & Health Sciences, Washington, DC USA; 9 Division of Neonatal and Developmental Medicine, Stanford University School of Medicine, Palo Alto, CA USA; 10 Division of Neonatology, Department of Pediatrics, Children’s Hospital Los Angeles, Keck School of Medicine, University of Southern California, Los Angeles, CA USA; 11 Pediatrix Medical Group of San Antonio, San Antonio, TX USA; 12 Department of Pediatrics, University of California San Francisco, San Francisco, CA USA; 13 Department of Pediatrics, Washington University in St. Louis, St. Louis, MO USA; 14 Department of Epidemiology and Biostatistics, University of California San Francisco, San Francisco, CA USA; 15 Department of Neurology, University of California San Francisco, San Francisco, CA USA

## Abstract

**Background:**

Mild hypoxic-ischemic encephalopathy (HIE) is increasingly recognized as a risk factor for neonatal brain injury. We examined the timing and pattern of brain injury in mild HIE.

**Methods:**

This retrospective cohort study includes infants with mild HIE treated at 9 hospitals. Neonatal brain MRIs were scored by 2 reviewers using a validated classification system, with discrepancies resolved by consensus. Severity and timing of MRI brain injury (i.e., acute, subacute, chronic) was scored on the subset of MRIs that were performed at or before 8 days of age.

**Results:**

Of 142 infants with mild HIE, 87 (61%) had injury on MRI at median age 5 (IQR 4–6) days. Watershed (23%), deep gray (20%) and punctate white matter (18%) injury were most common. Among the 125 (88%) infants who received a brain MRI at ≤8 days, mild (44%) injury was more common than moderate (11%) or severe (4%) injury. Subacute (37%) lesions were more commonly observed than acute (32%) or chronic lesions (1%).

**Conclusion:**

Subacute brain injury is common in newborn infants with mild HIE. Novel neuroprotective treatments for mild HIE will ideally target both subacute and acute injury mechanisms.

**Impact:**

- Almost two-thirds of infants with mild HIE have evidence of brain injury on MRI obtained in the early neonatal period.
- Subacute brain injury was seen in 37% of infants with mild HIE.
- Neuroprotective treatments for mild HIE will ideally target both acute and subacute injury mechanisms.

## Introduction

Hypoxic-ischemic encephalopathy (HIE), an important cause of neonatal encephalopathy (NE), affects approximately 1.5 out of every 1000 live births^1^ and is a major cause for developmental disability in children, leading to cerebral palsy, seizures, and cognitive impairment. Severity of HIE is clinically evaluated, commonly using the Sarnat grading system^2^. Neonates with mild encephalopathy by Sarnat grading are considered to have a more favorable neurodevelopmental prognosis^3^. For this reason, randomized controlled trials testing therapeutic hypothermia^4,5^ (TH) and other neuroprotective therapies for HIE^6,7^ have excluded mild HIE patients.

In recent years, mild HIE has been increasingly linked to adverse outcomes including brain injury and neurodevelopmental impairment. For instance, recent studies suggest that 38–61%^8–13^ of neonates with mild HIE have abnormal brain magnetic resonance imaging (MRI), a similar proportion as described in the moderate to severe HIE population. Furthermore, a recent meta-analysis of functional outcomes identified neurodevelopmental impairment in up to 25% of infants with mild HIE^14^.

The timing of brain injury in infants with mild HIE has not been well studied. Whether the brain injury seen in infants with mild HIE occurred remote from the time of birth allowing the infant time to partially recover by the time of delivery is unknown. A better understanding of the timing and pattern of brain injury in mild HIE may help inform future studies of novel treatments. We examined the timing, pattern, and severity of MRI brain injury in a multicenter cohort of infants with mild HIE.

## Methods

In this retrospective cohort study, we identified term and near-term infants born at ≥36 weeks gestational age at nine U.S. hospitals in 2013–2019 who were diagnosed with mild HIE. We evaluated all newborn infants with HIE at these hospitals in 2017–2019 for potential enrollment in *High-dose Erythropoietin for Asphyxia and Encephalopathy* (HEAL)^7^, a phase 3 trial investigating the efficacy of erythropoietin as a neuroprotective therapy for moderate to severe HIE. Infants were assessed using a modified Sarnat examination^7^ by trained examiners. Infants with mild HIE were excluded from the HEAL Trial and were instead enrolled in the current observational study. During the years 2013–2015, five of the sites had systematically identified infants with mild HIE using the same Sarnat criteria for a phase 2 clinical trial of erythropoietin for HIE^15^; the infants with mild HIE who were excluded from the phase 2 trial were also included in the current study. We defined mild HIE as at least one Sarnat exam abnormality of any severity in any of the six categories (i.e., consciousness, activity, tone, posture, primitive reflexes, or autonomic nervous system) documented at any time between 1–6 h of age^16^, but fewer than three moderate/severe Sarnat abnormalities as required to meet the definition of moderate to severe HIE^4,5^. Additionally, infants had evidence of perinatal depression with at least one of the following: 10-minute Apgar score <5, need for resuscitation at 10 min, pH < 7.00 in an arterial or venous cord or infant gas performed by 60 min of age, or base deficit ≥15 mmol/L in a cord or infant gas performed by 60 min of age. We included infants regardless of whether they were born at the participating hospital or transferred from an outside hospital for tertiary care. Patients were excluded from the study if any of the following were present: birthweight <1800 g (i.e., intrauterine growth restriction), head circumference <30 cm, or encephalopathy occurring after birth (i.e., post-natal collapse).

For all infants with mild HIE, we determined the following from medical record review: gestational age; birthweight; clinical diagnosis of chorioamnionitis; sentinel event defined as placental abruption, uterine rupture, shoulder dystocia, prolapsed cord or tight nuchal cord; treatment with TH; and length of hospital stay.

Study subjects received a brain MRI using local neuroimaging protocols as part of clinical care. These neuroimaging studies were performed on MRIs from three manufacturers: General Electric (46%), Siemens (34%) and Philips (20%). Most studies (78%) were performed at 3 T magnetic field strength, while the remaining were performed at 1.5 T. In infants who were treated with TH, the brain MRI was typically performed after rewarming (i.e., at 4–6 days of age). All subjects who did not receive TH were enrolled at a single hospital (UTSW) where TH is not used to treat mild HIE; these subjects also received a brain MRI as part of their routine clinical care.

**Fig. 1:**
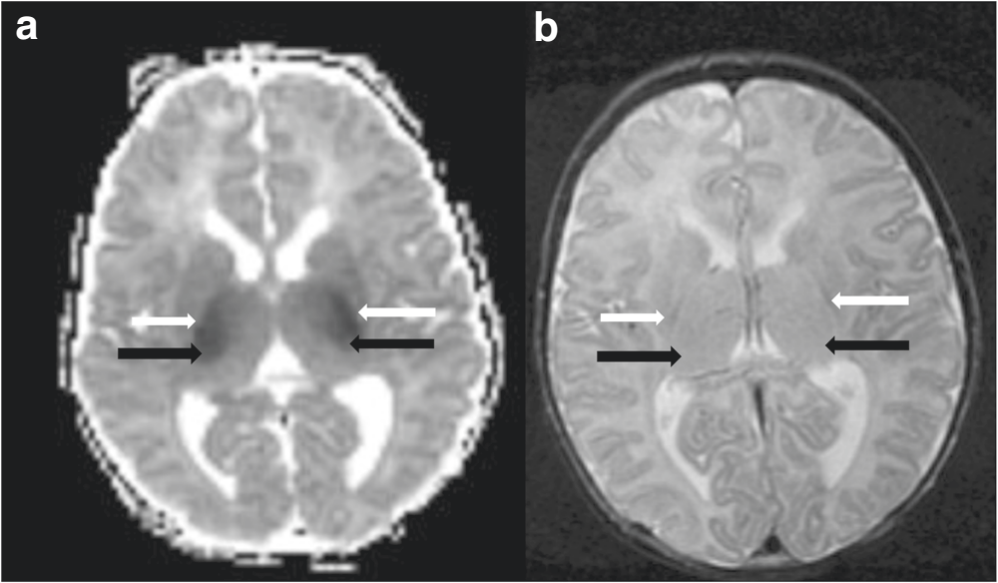
Acute, deep gray pattern of hypoxic ischemic brain injury. **a** ADC map demonstrating restricted diffusion involving the posterior limbs of the internal capsules (white arrows) and ventrolateral thalami (black arrows) and **b** T2-weighted imaging demonstrating hyperintensity involving the posterior limb of the internal capsule (white arrows) and bilateral thalami (black arrows).

**Fig. 2:**
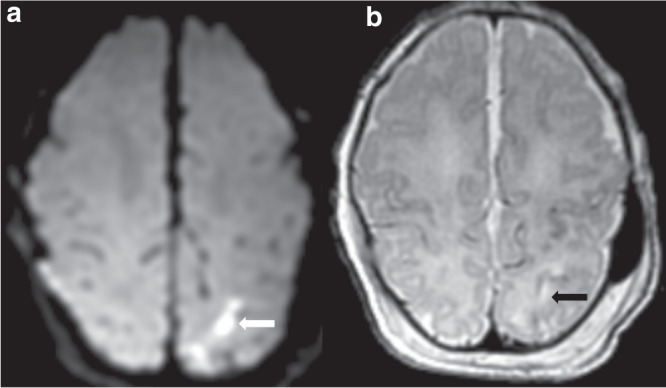
Acute arterial watershed pattern of hypoxic ischemic brain injury. **a** DWI demonstrating restricted diffusion (white arrow) and **b** T2 demonstrating hyperintensity (black arrow) in the left superior parietal white matter.

**Fig. 3:**
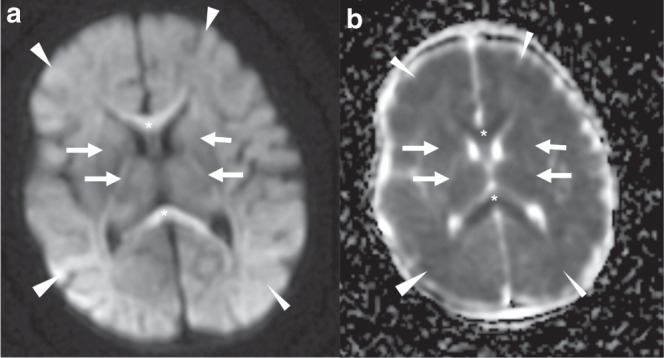
Acute, severe deep gray and watershed pattern of injury. DWI (**a**) and ADC map (**b**) demonstrate restricted diffusion involving the deep gray (white arrows denoting reduced diffusion in the putamen, globi paladi, ventrolateral thalamus, posterior limb of the internal capsule) and watershed pattern (white arrowheads denoting reduced diffusion involving the subcortical white matter), with global injury score 84. Additional reduced diffusion involving the genu and splenium of the corpus callosum (asterisks) is compatible with pre-Wallerian degeneration from intramyelinic edema.

**Fig. 4:**
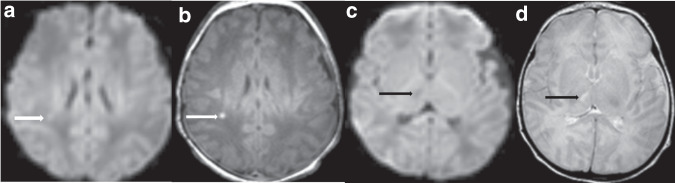
Multiple injury patterns and acuities of injury. Acute punctate white matter injury (white arrows) with reduced diffusion (**a**) and T1 hyperintensity (**b**). Additional subacute injury to the right thalamus (black arrows) with no reduced diffusion (**c**) and T2 hyperintensity (**d**), and without volume loss.

Brain MRIs were scored independently by 2 reviewers with extensive experience using a validated HIE severity classification system^17^; discrepancies were then resolved by consensus among all four members of the neuroimaging core (Y.L., R.M., J.W., A.M.). The MRI scoring system assigns a severity score based on extent of signal abnormality in specified regions of the brain on T1, T2, and DWI sequences. The global injury score (range 0–138) is the sum of all regional numeric injury scores. Severity of injury was pre-defined as: 0 = no injury; 1–11 = mild injury; 12–32 = moderate injury; 33–138 = severe injury^17^. The quality of the imaging with respect to motion artifact was recorded on a four-point scale: none; mild (unlikely to impact interpretation), moderate (may impact interpretation), and severe (obfuscates interpretation). A global injury severity score was calculated only in patients imaged within 8 days of age, because diffusion-weighted signal abnormalities, which constitute a component of the scoring system, typically disappear or undergo “pseudonormalization” by approximately 8 days of age^18,19^. Examples of different patterns and acuities of injury are provided in Figs. 1–4.

Each patient was classified as having one or more of the following patterns of brain injury: none, watershed, deep gray, punctate white matter lesions, arterial ischemic stroke, focal parenchymal lesions, hippocampal injury, and atypical lesions. Atypical lesions were defined as any abnormality not fitting into the previously described categories, and thus are atypical findings in the setting of HIE. Intraparenchymal and subdural hemorrhages were scored as trace, mild/moderate if no mass effect, and severe if mass effect was present. Intraventricular hemorrhage was similarly classified as trace, mild/moderate if no ventricular dilation, and severe if ventricular dilation was present. Subarachnoid hemorrhage was scored as either trace or focal.

The time window of brain injury was assessed in all brain MRI studies performed within 8 days of age. Acute injury was defined by the presence of restricted diffusion. Subacute injury was defined by presence of an abnormality on T1 and T2-weighted imaging without accompanying diffusion abnormality or volume loss. Chronic injury was defined by the presence of parenchymal volume loss.

We examined how brain injury timing and severity varied by clinical factors such as antenatal complications and treatment with TH using Wilcoxon Rank-Sum test for continuous variables and Pearson’s chi-squared test for categorical variables. We also examined whether the frequency of brain injury varied by the number of Sarnat exam abnormalities. Finally, we compared the rate of MRI brain injury in subjects who met criteria for mild HIE based on more restrictive definitions used in two clinical trials: (1) MEND (NCT03071861): mild HIE = one or two moderate or severe modified Sarnat abnormality in any of the six categories^20^; and (2) TIME (NCT04176471): mild HIE = at least 2 Sarnat abnormalities of any severity, but not qualifying for moderate/severe encephalopathy. This study was approved by the Institutional Review Boards at all participating institutions.

## Results

We identified 149 infants with mild HIE, 7 of whom were excluded due to unavailable MRI (5) or MRI that was not interpretable due to severe motion degradation (2). The remaining 142 infants comprise the study population. Brain MRIs were performed at a median age of 5 days (interquartile range (IQR) 4–6) and 70% had no motion, 23% had mild motion, and 7% had moderate motion on T1, T2, or DWI sequences. Infants with mild HIE exhibited significant cord blood acidosis and depressed Apgar scores (Table 1). Both clinical chorioamnionitis and sentinel events were present in about one-third of subjects.

**Table 1:** Clinical features of 142 infants with mild HIE stratified by presence of brain injury on MRI.

	All	Brain injury	No brain injury	
	*N* = 142	*N* = 87	*N* = 55	*p* value
Gestational age (mean, weeks)	39.2	39.1	39.4	0.25
Birthweight (mean, grams)	3292 g	3295	3288	0.93
Male	64%	70%	55%	0.06
Born at outside hospital	95 (67%)	57 (66%)	38 (69%)	0.66
Lowest cord pH (mean)	7.00	7.00	7.01	0.91
5-min Apgar score (median, range)	5 (0–9)	4.5 (0–9)	4 (1–9)	0.28
10-min Apgar score (median, range)	6 (1–9)	6 (1–9)	6 (1–9)	0.34
5 or greater Sarnat abnormalities	62%	61%	64%	0.75
Therapeutic Hypothermia	85%	83%	89%	0.30
Clinical chorioamnionitis	35%	35%	35%	0.99
Sentinel events	34%	36%	33%	0.72
Length of hospitalization (median days, IQR)	8 (6–13)	9 (7–14)	8 (6–12)	0.64

Brain parenchymal injury was present in 87 (61%) infants with mild HIE. No clinical factors were significantly associated with presence of brain injury (Table 1). The most common brain injury patterns were watershed (22%), deep gray nuclei (20%), punctate white matter (18%), and atypical lesions (18%, Table 2). Twenty-two percent had more than one injury pattern. Among the 142 infants who met our study inclusion criteria, 127 (89%) also met the TIME study definition, and 134 (94%) met the MEND^20^ study definition of mild HIE. The frequency of brain injury was unchanged when we analyzed only infants who met these more restrictive criteria (60% using TIME study definition; 61% using MEND study definition).

**Table 2:** Clinical features and MRI patterns of brain injury in infants with mild HIE who received therapeutic hypothermia, compared to those who did not receive therapeutic hypothermia.

	All *N* = 142	Therapeutic hypothermia *N* = 121	No therapeutic hypothermia *N* = 21	*P* value
Clinical features				
Gestational age (mean, weeks)	39.2	39.3	38.8	0.92
Birthweight (mean, grams)	3292	3389	3276	0.41
Male	91 (64%)	17 (81%)	74 (61%)	0.08
Lowest cord pH (mean)	7.0	7.0	7.0	0.57
5-minute Apgar (median, range)	5 (3–6)	4 (3–6)	7 (5–8)	<0.0001
10-minute Apgar (median, range)	6 (5–7)	6 (5–7)	6 (5–8)	0.54
Brain MRI				
Age at scan (median, days)	5 (4–6)	5 (4–7)	4 (3–4)	<0.001
Brain injury present	87 (61%)	72 (60%)	15 (71%)	0.30
Pattern of injury				
Normal	55 (39%)	49 (40%)	6 (29%)	0.30
Watershed	32 (23%)	22 (18%)	10 (48%)	0.002
Deep gray	28 (20%)	24 (20%)	4 (19%)	0.92
Punctate white matter	24 (17%)	19 (16%)	5 (24%)	0.48
Focal parenchymal	12 (8%)	10 (8%)	2 (10%)	0.95
Arterial ischemic stroke	5 (4%)	4 (3%)	1 (5%)	0.62
Hippocampal	2 (1%)	2 (2%)	0 (0%)	0.53
Atypical^a^	27 (19%)	25 (21%)	2 (10%)	0.29
Multiple patterns	31 (22%)	26 (21%)	5 (24)%	0.26

^a^Atypical patterns included: other supratentorial white matter signal abnormality (*N* = 8), cerebellar white matter signal abnormality (*N* = 5), callosal and/or anterior commissural reduced diffusion in the absence of other deep gray or white matter signal abnormality (*N* = 5), cerebellar hemorrhage (*N* = 2), signal abnormality suggestive of kernicterus (*N* = 2).

Intracranial hemorrhage was present in 60 (42%) infants, including 21 (15%) with moderate or severe hemorrhage. The most common location of hemorrhage was subdural (34%), followed by intraventricular (12%), intraparenchymal (6%), and subarachnoid (3%). Three subdural hemorrhages were classified as large enough to exhibit mass effect and one large intraventricular hemorrhage led to non-communicating hydrocephalus. There were no cases of large intraparenchymal hemorrhage.

The majority (85%) of infants in our cohort were treated with TH. The 21 infants with mild HIE who did not receive TH exhibited no difference in mean lowest cord pH when compared to the treated group (Table 2). Infants who did not receive TH had a higher median 5-min Apgar than those who did receive TH (7 vs. 4, *p* < 0.0001). Infants who underwent TH did not differ significantly from the untreated group in frequency of brain injury on MRI (60% vs. 71%, *p* = 0.30, Table 2). However, watershed injury was less common in those who received TH (18% vs. 48%, *p* = 0.003).

Among the 125 (88%) of infants with mild HIE who received an early brain MRI at ≤8 days of age, 75 (60%) had evidence of parenchymal brain injury. The median injury score in this group with early MRI was 5 (IQR 2–9, range 0–84) and when present, the brain injury was predominantly mild (Table 3). A greater percentage of infants exhibited subacute (37%) than acute (32%) or chronic (1%) injury. There were no clinical factors that were significantly associated with either severity or timing of brain injury in the subgroup of infants with early MRI.

**Table 3:** Injury severity and acuity among 125 infants with mild HIE who received a brain MRI at or before 8 days of age.

	All *N* = 125	Therapeutic hypothermia *N* = 104	No therapeutic hypothermia *N* = 21	*P* value
Injury score (median; IQR)	5 (2–9)	5 (2–9)	6 (2–10)	0.97
Injury severity				0.59
No injury	50 (40%)	44 (42%)	6 (29%)	
Mild	55 (44%)	43 (42%)	12 (57%)	
Moderate	14 (11%)	12 (12%)	2 (10%)	
Severe	5 (4%)	4 (4%)	1 (5%)	
Injury acuity				
No injury	50 (40%)	44 (42%)	6 (29%)	0.24
Acute	40 (32%)	34 (33%)	21 (29%)	0.71
Subacute	46 (37%)	36 (35%)	10 (48%)	0.26
Chronic	1 (1%)	1 (1%)	0 (0%)	0.65
Multiple acuities	18 (14%)	15 (14%)	3 (14%)	0.76

## Discussion

In this large multicenter study designed to evaluate the timing and pattern of brain injury in infants with mild HIE, we found that about two-thirds of infants had evidence of brain injury on MRI. The high rate of subacute lesions in our cohort is a novel finding, as few studies have evaluated the timing of brain injury in mild HIE. Additionally, we found that variations in the definition of mild HIE have minimal impact on the observed frequency of brain injury.

Among infants with mild HIE, 39% exhibited subacute brain injury defined as T1 or T2 signal abnormality without restricted diffusion to signify acute injury, and without volume loss to signify chronic injury. As we excluded infants imaged after 8 days of age from this analysis, this relatively high rate of subacute signal abnormality cannot be explained by pseudonormalization of diffusion abnormalities. Acute injury was also common, occurring in 31% of subjects. Although a lack of diffusion restriction within an area of brain injury suggests that the injury occurred at least over a week before the time of imaging, it is not possible to pinpoint the exact time of hypoxic-ischemic brain injury based on neuroimaging. However, the large percentage of infants with subacute injury in our study raises the possibility that mild encephalopathy may in many cases reflect injury that occurred early enough to provide the infant time to partially recover by the time of delivery, and thus to exhibit milder signs of encephalopathy.

The issue of timing of injury is an important consideration when designing treatments for HIE. The intracellular mechanisms of hypoxic-ischemic brain injury are complex and evolve over time^21^. Following a primary phase of neuronal cell death, many surviving cells will partially recover during a latent phase which provides a window of opportunity to intervene with therapies such as TH^22^. The latent phase is then followed by a secondary phase of cell death due to numerous intracellular mechanisms such as apoptosis, inflammation and mitochondrial failure^22^. It is known that TH is an effective therapy for reducing the risk of death or neurologic disability in infants with moderate to severe HIE^23^. However, no randomized controlled trials have tested the efficacy of TH for treating milder cases of HIE, and observational data supporting the use of this treatment for mild HIE show inconsistent results^13,24^. The frequent subacute injury observed in our cohort raises the possibility that TH may be less neuroprotective in infants with mild HIE, given that the baby may be born beyond the neuroprotective window for TH to be effective, namely six hours after injury. However, this hypothesis remains a speculation and studies evaluating the efficacy of TH for mild HIE are urgently needed.

The 61% rate of MRI brain injury among all subjects in our cohort is consistent with previous estimates of the frequency of brain injury in mild HIE (38–61%)^8–13^. White matter injury in an arterial watershed distribution was the most common pattern of brain abnormality seen in our cohort, a finding that is also consistent with previous studies of mild HIE^8,11,12,25–29^. The predominance of mild severity of brain injury is also consistent with previous reports^9,11^.

Whereas infants with mild HIE were previously thought to have an excellent neurologic prognosis, a recent review suggests that up to 25% of infants with mild HIE are at risk for neurodevelopmental deficits in infancy or childhood^14^. In the PRIME prospective observational study, for instance, 16% of infants with mild HIE had disability at 18–22 months of age^28,30^. There has been a significant therapeutic drift in recent years with many hospitals offering TH to infants with mild HIE, because we lack other neuroprotective therapies for this condition, and because the evolving nature of the infant exam can make it challenging to establish the severity of HIE with certainty. A recent survey of practices in the United Kingdom found that 76% of centers that offer TH for moderate to severe HIE also provide this therapy to infants with mild HIE^31^, a practice that is shared by eight of our nine study sites.

It is important to note that our observational study was not designed to determine the efficacy of TH for mild HIE. Although the rate of brain injury was no different in the group that did and did not receive TH, we did observe a significantly lower frequency of watershed injury in the TH group, a finding that has been reported in other observational cohorts^13,25,32^. The lower rate of watershed injury suggests a possible benefit to the TH, since selection bias resulting from sicker infants being preferentially chosen to receive TH should make it more difficult to appreciate MRI improvements among infants who received TH. However, conclusions regarding the potential benefit of TH for mild HIE will require well-designed clinical trials or comparative efficacy studies that are specifically designed to evaluate this question.

Prior studies have found that perinatal sentinel events such as placental abruption or uterine rupture are present in about one-third of cases of moderate to severe HIE^33,34^, which is similar to the 34% rate of sentinel events seen in our cohort. In our cohort, 70% of male infants had brain injury on MRI, compared to 55% of female infants. Although this difference was not statistically significant (*P* = 0.06), it may reflect the sexual dimorphism that has been well described in HIE^35^.

A challenge faced by researchers studying mild HIE has been the lack of a universally accepted set of diagnostic criteria^9,20,28^. Our study found that the rate and severity of brain injury did not vary significantly when we applied two additional study definitions of mild HIE that are currently being used in research settings. These findings are reassuring and suggest that the lack of a consensus definition of mild HIE is unlikely to cause large discrepancies in neuroimaging findings or rates of brain injury.

Our study has several strengths. In addition to its large size, we identified subjects in a systematic fashion at multiple tertiary care centers, thus increasing generalizability. Almost all subjects received a brain MRI within the first 8 days of age, allowing us to evaluate the timing of brain injury in mild HIE. However, our study has important limitations. Because of the observational nature of the study, the fact that only a small number of infants did not receive TH, and because all infants who did not receive TH were enrolled at a single site, we are unable to determine whether TH improved neuroimaging outcomes in our cohort. The study further is limited by potential selection bias since hospitals that performed TH in neonates with mild HIE may have selected more severely affected infants in the mild HIE spectrum to receive this treatment, thus artificially increasing the rate of brain MRI abnormalities in our cohort. Our study lacked harmonization of MRI protocols across institutions and platforms. We lacked information regarding aEEG findings which could be used to confirm the presence of mild HIE, and the study also does not include neurodevelopmental outcomes. Finally, although we infer that T2 signal abnormality in a pattern compatible with HIE likely represents subacute injury, we cannot exclude the possibility that some of these lesions may also represent areas of inflammation.

In conclusion, we found that subacute brain injury was common in infants with mild HIE. Novel neuroprotective therapies will ideally target both subacute and acute injury mechanisms. The large number of infants with subacute injury suggests that mild HIE may be the result of injury that occurred hours or days prior to delivery. Future clinical trials are needed to evaluate the efficacy of TH and other therapeutic agents^20^ as a treatment for infants with mild HIE, and to determine which subset of these patients will most likely benefit from these treatments.

## Data Availability

The datasets generated during and/or analyzed during the current study are available from the corresponding author on reasonable request.

## References

[1] Kurinczuk JJ, White-Koning M, Badawi N (2010). Epidemiology of neonatal encephalopathy and hypoxic-ischaemic encephalopathy. Early Hum. Dev..

[2] Sarnat HB, Sarnat MS (1976). Neonatal encephalopathy following fetal distress: a clinical and electroencephalographic study. Arch. Neurol..

[3] Robertson C, Finer N (1985). Term infants with hypoxic-ischemic encephalopathy: outcome at 3.5 years. Dev. Med. Child Neurol..

[4] Azzopardi DV (2009). Moderate hypothermia to treat perinatal asphyxial encephalopathy. N. Engl. J. Med..

[5] Shankaran S (2005). Whole-body hypothermia for neonates with hypoxic-ischemic encephalopathy. N. Engl. J. Med..

[6] Wu YW (2016). High-dose erythropoietin and hypothermia for hypoxic-ischemic encephalopathy: a phase II trial. Pediatrics.

[7] Juul SE (2018). High-dose erythropoietin for asphyxia and encephalopathy (HEAL): a randomized controlled trial-background, aims, and study protocol. Neonatology.

[8] Gagne-Loranger M, Sheppard M, Ali N, Saint-Martin C, Wintermark P (2016). Newborns referred for therapeutic hypothermia: association between initial degree of encephalopathy and severity of brain injury (What about the newborns with mild encephalopathy on admission?). Am. J. Perinatol..

[9] Walsh BH (2017). The frequency and severity of magnetic resonance imaging abnormalities in infants with mild neonatal encephalopathy. J. Pediatr..

[10] Massaro AN (2015). Short-term outcomes after perinatal hypoxic ischemic encephalopathy: a report from the Children’s Hospitals Neonatal Consortium HIE focus group. J. Perinatol..

[11] Rao R (2019). Neurodevelopmental outcomes in neonates with mild hypoxic ischemic encephalopathy treated with therapeutic hypothermia. Am. J. Perinatol..

[12] Rao R (2022). Utilization of therapeutic hypothermia and neurological injury in neonates with mild hypoxic-ischemic encephalopathy: a report from Children’s Hospital Neonatal Consortium. Am. J. Perinatol..

[13] Montaldo P (2019). Therapeutic hypothermia initiated within 6 h of birth is associated with reduced brain injury on MR biomarkers in mild hypoxic-ischaemic encephalopathy: a non-randomised cohort study. Arch. Dis. Child Fetal Neonatal Ed..

[14] Conway JM, Walsh BH, Boylan GB, Murray DM (2018). Mild hypoxic ischaemic encephalopathy and long term neurodevelopmental outcome - a systematic review. Early Hum. Dev..

[15] Wu YW (2016). High-dose erythropoietin and hypothermia for hypoxic-Ischemic encephalopathy: a phase II trial. Pediatrics.

[16] Chalak LF (2014). Neurodevelopmental outcomes after hypothermia therapy in the era of Bayley-III. J. Perinatol..

[17] Trivedi SB (2017). A validated clinical MRI injury scoring system in neonatal hypoxic-ischemic encephalopathy. Pediatr. Radiol..

[18] Bednarek N (2012). Impact of therapeutic hypothermia on MRI diffusion changes in neonatal encephalopathy. Neurology.

[19] McKinstry RC (2002). A prospective, longitudinal diffusion tensor imaging study of brain injury in newborns. Neurology.

[20] DuPont TL (2021). Darbepoetin as a neuroprotective agent in mild neonatal encephalopathy: a randomized, placebo-controlled, feasibility trial. J. Perinatol..

[21] Davidson JO, Gonzalez F, Gressens P, Gunn AJ (2021). Update on mechanisms of the pathophysiology of neonatal encephalopathy. Semin. Fetal Neonatal Med..

[22] Drury PP, Gunn ER, Bennet L, Gunn AJ (2014). Mechanisms of hypothermic neuroprotection. Clin. Perinatol..

[23] Tagin MA, Woolcott CG, Vincer MJ, Whyte RK, Stinson DA (2012). Hypothermia for neonatal hypoxic ischemic encephalopathy: an updated systematic review and meta-analysis. Arch. Pediatr. Adolesc. Med..

[24] Kariholu U (2018). Therapeutic hypothermia for mild neonatal encephalopathy: a systematic review and meta-analysis. Arch. Dis. Child Fetal Neonatal Ed..

[25] Goswami IR (2020). Characteristics and short-term outcomes of neonates with mild hypoxic-ischemic encephalopathy treated with hypothermia. J. Perinatol..

[26] Lally PJ (2014). Neonatal encephalopathic cerebral injury in South India assessed by perinatal magnetic resonance biomarkers and early childhood neurodevelopmental outcome. PLoS ONE.

[27] van Kooij BJM (2010). Serial MRI and neurodevelopmental outcome in 9- to 10-year-old children with neonatal encephalopathy. J. Pediatr..

[28] Prempunpong C (2018). Prospective research on infants with mild encephalopathy: the PRIME study. J. Perinatol..

[29] Dupont TL (2013). Short-term outcomes of newborns with perinatal acidemia who are not eligible for systemic hypothermia therapy. J. Pediatr..

[30] Chalak LF (2018). Prospective research in infants with mild encephalopathy identified in the first six hours of life: neurodevelopmental outcomes at 18–22 months. Pediatr. Res..

[31] Oliveira V (2018). Therapeutic hypothermia in mild neonatal encephalopathy: a national survey of practice in the UK. Arch. Dis. Child Fetal Neonatal Ed..

[32] Goswami IR (2020). Characteristics and short-term outcomes of neonates with mild hypoxic-ischemic encephalopathy treated with hypothermia. J. Perinatol..

[33] Parker S-J, Kuzniewicz M, Niki H, Wu YW (2018). Antenatal and intrapartum risk factors for hypoxic-ischemic encephalopathy in a US birth cohort. J. Pediatr..

[34] Nelson KB (2012). Antecedents of neonatal encephalopathy in the Vermont Oxford Network Encephalopathy Registry. Pediatrics.

[35] Rosenkrantz, T. S., Hussain, Z., Fitch, R. H. Sex differences in brain injury and repair in newborn infants: clinical evidence and biological mechanisms. *Front. Pediatr.* **7**. https://www.frontiersin.org/article/10.3389/fped.2019.00211 (2019). 10.3389/fped.2019.00211 PMC6606734 31294000

